# Acceptance and attitude of Lebanese lawyers and medical students toward surrogate pregnancy: a cross-sectional study

**DOI:** 10.1186/s12978-023-01638-4

**Published:** 2023-06-21

**Authors:** Rashad Nawfal, Jad Kassem, Lea Nicole Sayegh, Antony Haddad, Marly Azzi, Pascale Salameh, Lubna Tarabey, Fadi Abou-Mrad

**Affiliations:** 1 Division of Medical Ethics, Faculty of Medical Sciences, Lebanese University, Beirut, Lebanon; 2 Faculty of Medicine, American University of Beirut, Beirut, Lebanon; 3 Épidémiologie Clinique et Toxicologie-Liban, INSPECT-LB: Institut National de Santé Publique, Beirut, Lebanon; 4 Department of Primary Care and Population Health, University of Nicosia Medical School, Nicosia, Cyprus; 5 Faculty of Pharmacy, Lebanese University, Beirut, Lebanon; 6 Memory Clinic, Division of Neurology, Saint Charles Hospital, Fayadieh, Lebanon

**Keywords:** Ethics, Law, Surrogacy, Pregnancy, Lawyers, Medical Students

## Abstract

**Background:**

Little is known about the acceptance of specific populations of decision makers in Lebanon regarding surrogacy. This study aimed to explore the acceptance and attitude of Lebanese Lawyers and Medical Students regarding surrogacy.

**Methods:**

In total 248 medical students and 204 lawyers completed a questionnaire to assess socio-demographic data, attitude toward surrogacy, and three different clinical scenarios to assess patterns of thinking. Finally, we validated a scale to assess the acceptance of surrogacy in these two populations.

**Results:**

Concerning medical students, 54.8% reported they were supportive of surrogacy, 35.1% were neutral and 10.1% were against. For lawyers, 52.9% were supportive, 25% were neutral and 22.1% were against. Lawyers were more likely to be against surrogacy (p = 0.001).

After conducting a multivariate analysis on the whole studied population to find predictors of acceptance of surrogacy, the best predictors were being single (OR 0.415; 95% CI 0.228, 0.753; p < 0.01), a supportive reported attitude regarding surrogacy (OR 5.464; 95% CI 3.65, 8.13; p < 0.001) and believing that surrogacy is a solution worth discussing in Lebanon (OR 4.186; 95% CI 1.709, 10.256; p < 0.01).

Concerning the clinical scenarios, they showed that lawyers were more likely to oppose abortion regardless of the reason (p < 0.01). Also, in a case of gestational surrogacy, lawyers were more likely to give the right to the gestational carrier to keep the baby compared to medical students (p < 0.001).

**Conclusion:**

In conclusion, this study shows that only a minority of medical students and lawyers in Lebanon oppose surrogate pregnancy which warrants exploration of the perspective of other populations of decision makers in Lebanon to better guide legislations.

## Introduction

Infertility is a fairly common problem worldwide, affecting between 8 to 12% of couples of reproductive age [1]. Infertility is marked by the inability to achieve clinical pregnancy after 12 months of unprotected and regular sexual intercourse [1]. Female patients who endure infertility problems are more likely to develop stress, depression and anxiety [2]. When medical treatment fails to find solutions for infertility, Assisted Reproductive Technologies (ARTs) can be suggested to help infertile women get pregnant. The application of ARTs however has sparked ethical, religious, legal, and social controversies [3–5]. One of these widely debated ARTs is the use of third-party or gestational surrogacy. In some instances, prospective parents resort to traditional surrogacy, which has added layers of legal and social complications.

Surrogate pregnancy implies that a third party becomes pregnant, and then hands over the child to the couple [6]. There are 2 types of surrogacies: the first is gestational or full surrogacy whereby the parents’ embryo, a product of the intended father’s sperm and intended mother’s egg, is implanted in the surrogate uterus, whereas the second type is traditional surrogacy involving artificial insemination using the intended father’s or a donor’s sperm and the surrogate’s egg [7].

Regulations and laws that control this practice differentiate between the types of surrogacies as it could be either commercial in which the surrogate mother is compensated financially for the act [8] or altruistic in which the surrogate mother is simply reimbursed for medical and other reasonable expenses related to the pregnancy [9]. Religion has a considerable impact on people’s viewpoint toward surrogacy. Results of surveys done in Jordan that assessed the attitude of medical and paramedical students toward surrogate pregnancy showed a hesitancy to accept surrogacy mainly due to religious considerations [10, 11].

In Lebanon, ARTs are becoming increasingly accessible, but couples who are considering surrogacy are obliged to look overseas for their solution due to religious and legislative obstacles [12]. As in many countries in the Middle East, surrogate pregnancy in Lebanon remains illegal. The Islamic Republic of Iran remains the only country in the Middle East to legalize surrogacy [13], this is because jurisprudence is based on Islamic Sunni Sharia in most Middle Eastern countries while Iran follows Shiite sharia law [14].

Surrogate pregnancy is not only a solution for infertile women but also a means considered by gay couples to conceive a baby that in some instances has one of the parents’ genes, and by those who suffer from comorbidities that make them unable to complete their pregnancy.

Despite surrogate pregnancy being a very hot topic for debate, to the best of our knowledge no previous investigations were made in Lebanon to assess the point of view of specific stakeholders regarding surrogate pregnancy. Due to this lack of literature, we decided to assess in this study the point of view of lawyers and medical students who, in a way, are or will be key decision makers on this widely debatable topic.

The main objective of this study was to assess the perspective of Lebanese Lawyers and Medical students toward surrogate pregnancy, as well as to find socio-demographic data and points of view that could correlate with attitudes and perspectives toward surrogate pregnancy.

## Methods

### Study design

This is a cross-sectional, observational study. All medical students pursuing their final four years at any of the seven medical schools in Lebanon were included. All lawyers enrolled in the Beirut Bar Association, and currently practicing law in Lebanon were included. Lawyers enrolled in the Beirut Bar Association but practicing exclusively outside Lebanon were excluded to decrease the probability of having confounding factors.

Lawyers and medical students were recruited from different areas in Lebanon using social media platforms and phone calls. For Lawyers, the contact details of all officially registered lawyers were taken from the Beirut Bar Association Website [15]. Medical Students were contacted via their respective class social media chatting groups in which all students are participants. Both populations were asked to fill the questionnaire after completing the consent form via Google Forms survey software.

Data collection took place between April 2021 and August 2021 for both populations. The English version of the questionnaire was completed by Medical Students. Concerning lawyers, the English questionnaire as well as the consent form were translated by three different professional translators, then all three versions were matched, and the Arabic translation was administered to this population.

The questionnaire consisted of four parts: the first part asked about socio-demographic data, the second about perspective regarding surrogate pregnancy and highlights the perception toward the possible use of surrogacy for financial gain in Lebanon, the third assessed the opinion of participants concerning three different hypothetical cases of gestational and traditional surrogacy, and who should get the right to choose when performing an abortion. Finally, the fourth part assessed the attitude regarding surrogacy using a modified version of the Gestational Surrogacy Attitudes Scale (GSAS) [16] and validated to use on our population through Principal Component Analysis.

### Definitions

Socioeconomic Status was estimated through the ratio between the number of persons (including the participant) living in the same house, and the number of rooms in this house (excluding bathrooms and kitchen), person to room ratio was used as follows: High socioeconomic status for a person to room ratio less than 1, moderate socio-economic status for a person to room ratio equal to 1, and low socio-economic status for a person to room ratio more than 1.

### Validation and reliability of the scale

The Gestational Surrogacy Attitudes Scale (GSAS) was used for both populations to assess the attitude toward surrogacy. As the attitude becomes more positive, the total score will be higher. This is a 5-point Likert scale composed of 8 items which were scored as follows: I strongly agree = 5, I agree = 4, I am indecisive = 3, I disagree = 2, and I strongly disagree = 1. Questions 6 to 8 of the scale are negatively phrased and were reversed when scoring.

Validation of the scale was assessed by ways of face validity and construct validity. Face validity was established by the participants who stated they had no problem understanding and completing the questionnaire during the pilot phase of the study, as well as by experts in questionnaire formulation at the Medical Ethics Thesis Committee at the Lebanese University who approved the questionnaire to be administered. Construct validity was assessed through Principal Component Analysis, which yielded a Kaiser–Meyer–Olkin value of 0.887 and Bartlett’s test of sphericity that was significant (p < 0.001) indicating that the factor analysis is suitable. Also, inter-item correlation ranged between 0.518 and 0.857 which is acceptable (see Table 1).

**Table 1:** Factor coefficients for principal component analysis

	Component
Surrogacy is a good way to help infertile couples have a child with their own genetic characteristics	0.819
Surrogacy reduces psychological tension in infertile couples	0.843
Surrogacy improves the life satisfaction of infertile couples	0.857
Surrogacy can prevent divorce and strengthens family structure	0.734
If there is no other infertility treatment option, surrogacy could be the last choice	0.681
I prefer to be voluntarily childless rather than to accept surrogacy	0.723
Adoption is better than surrogacy	0.518
Surrogacy is against religion	0.588

Extraction method: principal component analysis

Reliability was established using Cronbach’s alpha on our final scale answers, a score of 0.7 and higher can be considered as acceptable [17]. Cronbach’s alpha for all survey answers in this study (n = 452) was equal to 0.866 indicating that the scale is reliable.

### Statistical analysis

Statistical analysis was carried out using the statistical software SPSS (Statistical Package for Social Sciences), version 22.0. Descriptive statistics were reported as mean, range and standard deviation (SD) for continuous variables, and as frequency and percentage for categorical variables. Concerning bivariate analysis, the *χ*^2^ test was used to detect an association between categorical variables, t-test or Mann–Whitney test was used to compare continuous variables. A multivariate analysis was also conducted to find predictors of a more positive attitude toward surrogacy. Statistical significance level was set at p < 0.05.

## Results

### Socio-demographic data

Of the 452 participants who participated in this study, 248 were medical students and 204 were lawyers.

Among medical students, 39.9% were male and 60.1% were female, mean age was 22.54 years of age, and standard deviation was 1.23 years. All medical students who participated in this study had no children and the vast majority 99.5% were not married.

For medical students, 59.3% had a high socioeconomic status, 20.6% had a moderate socioeconomic status, and 20.2% had a low socioeconomic status (see Table 2). Concerning the year of medical studies, 33.9% were 4th year medical students, 40.7% were 5th year medical students, 13.3% were 6th year medical students, and 12.1% were 7th year medical students (see Table 3).

**Table 2:** Socio-demographic data

	Study group		Total (N = 452)	P-value
	Medical students (N = 248)	Lawyers (N = 204)		
Nationality				
Lebanese	239	203	442	0.024
	96.4%	99.5%	97.8%	
Others	9	1	10	
	3.6%	0.5%	2.2%	
Age				
Mean (SD)	22.54 (1.23)	36.16 (10.23)	28.69 (9.69)	0.000
Min–Max	19–29	21–70	19–70	
Sex				
Male	99	82	181	0.952
	39.9%	40.2%	40.0%	
Female	149	122	271	
	60.1%	59.8%	60.0%	
Marital status				
Single	246	104	350	0.000
	99.2%	51.0%	77.4%	
Married	2	92	94	
	0.8%	45.1%	20.8%	
Divorced	0	8	8	
	0.0%	3.9%	1.8%	
How many children do you have?				
I have no children	248	125	373	0.000
	100.0%	61.3%	82.5%	
One child	0	23	23	
	0.0%	11.3%	5.1%	
2 to 4 children	0	56	56	
	0.0%	27.5%	12.4%	
Socioeconomic status				
Low	50	35	85	0.645
	20.2%	17.2%	18.8%	
Moderate	51	40	91	
	20.6%	19.6%	20.1%	
High	147	129	276	
	59.3%	63.2%	61.1%	

P < 0.05 is considered significant

**Table 3:** Year of medical studies

In what year of your medical studies are you currently enrolled?	Medical students
	N (%)
Med 1 (4th year)	84 (33.9)
Med 2 (5th year)	101 (40.7)
Med 3 (6th year)	33 (13.3)
Med 4 (7th year)	30 (12.1)

*Med 1* first year medical student; *Med 2* second year medical student; *Med 3* third year medical student; *Med 4* fourth year medical student

*N* Frequency, % Percentage

All 7 faculties of medicine in Lebanon were included in this study as follows, 32.3% study at the Lebanese University (LU), 16.1% study at the American University of Beirut (AUB), 9.7% study at Université Saint Joseph (USJ), 8.9% study at the Beirut Arab University (BAU), 2.8% study at the Lebanese American University (LAU), 6.5% study at the Université Saint Esprit de Kaslik (USEK) and 23.8% study at the University of Balamand (UOB) (see Table 4).

**Table 4:** Medical School of Participants

What university are you currently pursuing your medical studies in?	Medical students
	N (%)
LU	80 (32.3%)
UOB	59 (23.8%)
AUB	40 (16.1%)
USJ	24 (9.7%)
BAU	22 (8.9%)
USEK	16 (6.5%)
LAU	7 (2.8%)

LU, Lebanese University; UOB, University of Balamand; AUB, American University of Beirut; USJ, Université Saint Joseph; BAU, Beirut Arab University; USEK, Université Saint Esprit de Kaslik; LAU, Lebanese American University

N: Frequency, %: Percentage

Among lawyers, 40.2% were males and 59.8% were females, mean age was 36.16 years of age, and standard deviation was 10.23 years. 51% of lawyers were single, 45.1% were married, and 3.9% were divorced. The socioeconomic status was high in 63.2%, moderate in 19.6% and low in 18.8%. Socio-demographic data for both populations of medical students and lawyers was detailed in Table 2.

### Attitude toward surrogacy

Concerning the attitude toward surrogate pregnancy, of the 452 total participants in this study, 54% were supportive, 30.5% were neutral and 15.5% were against, and lawyers were more likely to be against this practice compared to medical students with p < 0.001. Of participants who stated that they are supportive or neutral, 44.2% were against commercialization of surrogate pregnancy, and 55.8% were supportive. Lawyers were more supportive of commercialization than medical students with p < 0.01.

Of participants against surrogate pregnancy, 27.1% would change their mind if surrogacy was the only means to have kids for a couple, and 72.9% would not change their mind when presented with this information. A full description of the attitude toward surrogacy can be found in Table 5.

**Table 5:** Attitude toward surrogate pregnancy

	Study group		Total (N = 452)	P value
	Medical students	Lawyers		
	(N = 248)	(N = 204)		
What is your attitude toward surrogate pregnancy?				
Supportive	136	108	244	0.001
	54.8%	52.9%	54.0%	
Neutral	87	51	138	
	35.1%	25.0%	30.5%	
Against	25	45	70	
	10.1%	22.1%	15.5%	
If your attitude is supportive or neutral, do you think surrogacy should be commercialized in Lebanon				
No	113	56	169	0.003
	50.7%	35.2%	44.2%	
Yes	110	103	213	
	49.3%	64.8%	55.8%	
If your attitude is against surrogate pregnancy, would you change your mind if you knew that surrogate pregnancy is the only means to have kids for a couple?				
No	15	36	51	0.071
	60.0%	80.0%	72.9%	
Yes	10	9	19	
	40.0%	20.0%	27.1%	
Attitude related to social values				
No	59	75	134	0.003
	23.8%	36.8%	29.6%	
Yes	189	129	318	
	76.2%	63.2%	70.4%	
Attitude related to religion				
No	187	162	349	0.312
	75.4%	79.4%	77.2%	
Yes	61	42	103	
	24.6%	20.6%	22.8%	
Attitude related to studies you read				
No	180	131	311	0.056
	72.6%	64.2%	68.8%	
Yes	68	73	141	
	27.4%	35.8%	31.2%	

p < 0.05 is considered statistically significant

Other questions concerning attitudes and knowledge regarding surrogate pregnancy included awareness about Lebanese couples who went for surrogacy as a solution for infertility, 85.6% responded they were not aware of this practice in Lebanon and 14.4% responded they were.

Concerning the belief that surrogacy is a solution worth discussing in Lebanon, 82.7% believe it is worth discussing and 17.3% believe it is not.

Regarding the knowledge about the position of their religion, 54% of all participants were aware of the religion’s position, with lawyers being more knowledgeable about this position compared to medical students (p < 0.001), and 41.2% would follow the position of their religion in this matter. A description of these attitudes and knowledge can be found in Table 6.

**Table 6:** Attitude and knowledge concerning surrogate pregnancy

	Study group		Total (N = 452)	P value
	Medical students (N = 248)	Lawyers (N = 204)		
Are you aware of any Lebanese couple who went for surrogacy as a solution for infertility?				
No	219	168	387	0.073
	88.3%	82.4%	85.6%	
Yes	29	36	65	
	11.7%	17.6%	14.4%	
Do you believe surrogacy is a solution worth discussing in Lebanon as a potential treatment for infertility?				
No	44	34	78	0.763
	17.7%	16.7%	17.3%	
Yes	204	170	374	
	82.3%	83.3%	82.7%	
Do you know the official position of your religion toward surrogate pregnancy?				
No	166	78	244	0.000
	66.9%	38.2%	54.0%	
Yes	82	126	208	
	33.1%	61.8%	46.0%	
Would you follow the official position of your religion toward surrogate pregnancy?				
No	138	128	266	0.127
	55.6%	62.7%	58.8%	
Yes	110	76	186	
	44.4%	37.3%	41.2%	

N, frequency; %, percentage

p < 0.05 is considered statistically significant

Additional questions assessed the attitude toward surrogate pregnancy and if it was related to social values, religion, or studies the participants read. These results can be found in Table 7.

**Table 7:** What is the attitude related to?

	Study group		Total (N = 452)
	Medical students (N = 248)	Lawyers (N = 204)	
Attitude related to social values			
No	59	75	134
	23.8%	36.8%	29.6%
Yes	189	129	318
	76.2%	63.2%	70.4%
Attitude related to religion			
No	187	162	349
	75.4%	79.4%	77.2%
Yes	61	42	103
	24.6%	20.6%	22.8%
Attitude related to studies you read			
No	180	131	311
	72.6%	64.2%	68.8%
Yes	68	73	141
	27.4%	35.8%	31.2%

p < 0.05 is considered statistically significant

### Clinical scenarios

Three clinical scenarios were also presented to our populations to assess the attitude toward gestational and traditional surrogacy as well as who should have the right to choose abortion in surrogate pregnancies.

The first clinical scenario asks, in a case of gestational surrogacy, if the surrogate mother can make the decision to keep the baby. 91.5% of medical students say she should give the baby back against her will and 8.5% said she should keep the baby, for lawyers, 75.5% said she should give the baby back against her will and 24.5% said she should keep the baby, thus lawyers were more likely to side with the surrogate mother to keep the baby (p < 0.001).

The second clinical scenario asks, in a case of traditional surrogacy, if the surrogate mother can make the decision to keep the baby. 39.9% of medical students answered she should give the baby back against her will and 60.1% said she should keep the baby, for lawyers, 31.4% said she should give the baby back against her will and 68.6% said she should keep the baby, the difference between the two populations was not significant, however, at p = 0.06.

Regarding the third clinical scenario which asked about who should have the right to make the decision for an abortion, 13.3% of medical students were against abortion in all cases compared to 27% of lawyers (p < 0.01). 8.5% of medical students said it would be the couple’s decision in all cases compared to 5.9% of lawyers, 10.5% of medical students said it would be the surrogate’s mother decision compared to 8.3% of lawyers and finally, 67.7% of medical students said the surrogate mother can decide only if her health is at risk compared to 58.8% of lawyers.

A full description of responses to the clinical scenarios can be found in Table 8.

**Table 8:** Clinical scenarios

Clinical scenario	Answer	Medical students (N = 248)	Lawyers (N = 204)	Total (N = 452)	p-value
A lady has normal eggs but cannot get pregnant. She and her husband decide to get a child through surrogate pregnancy, so they sign a contract with the surrogate mother that she would be paid a sum of money, conceive the child who is genetically the son of the husband and his wife, and return the child to his genetic parents Upon delivering the baby, the surrogate mother’s normal maternal instincts overwhelm her and she gets so attached to the baby she just gave birth to, and refuses to give the baby to his genetic parents. In your opinion, in a perfect world, the surrogate mother should:	Give the baby back against her will	227	154	381	0.000
		91.5%	75.5%	84.3%	
	Have the right to keep the baby and terminate the contract	21	50	71	
		8.5%	24.5%	15.7%	
What would your opinion be if, in the same case as before, the problem is with the wife’s eggs, and the surrogate mother also donated her egg as part of the contract. Which means that now, the surrogate mother is also at the same time genetically the mother of the baby Upon delivering the baby, the surrogate mother’s normal maternal instincts overwhelm her and she gets so attached to the baby she just gave birth to, and refuses to give the baby to his genetic parents. In your opinion, in a perfect world, the surrogate mother should:	Give the baby back against her will	99	64	163	0.060
		39.9%	31.4%	36.1%	
	Have the right to keep the baby and terminate the contract	149	140	289	
		60.1%	68.6%	63.9%	
What if the surrogate mother decides to abort the baby, as during a visit to the doctor, she found out that the baby suffers from a severe congenital disorder. Should she have the privilege to take such decision?	I am against abortion in all cases	33	55	88	0.003
		13.3%	27.0%	19.5%	
	No, it is the couple's decision in all cases	21	12	33	
		8.5%	5.9%	7.3%	
	The surrogate mother can decide only if her health is at risk if she continues with the pregnancy	168	120	288	
		67.7%	58.8%	63.7%	
	Yes, it is her body, she should have the right to decide in all cases	26	17	43	
		10.5%	8.3%	9.5%	

p < 0.05 is considered statistically significant

### Acceptance of surrogacy scale

Concerning the answers to the scale, for medical students, the mean for total scores was 27.87, standard deviation was 5.99 and range was 8–40. For lawyers, mean score was 27.8, standard deviation was 8.4 and range was 8–40. For both populations together, mean was 27.84, standard deviation was 7.17 and range was 8–40. A statistically significant difference between our two populations could only be found for one statement: “Surrogacy is a good way to help infertile couples have a child with their own genetic characteristics”, whereby medical students had a more positive attitude toward this statement with p < 0.05. The results to the scale answers can be found in Table 9.

**Table 9:** Acceptance of surrogacy scale

		Study group		Total (N = 452)	P value
		Medical students (N = 248)	Lawyers (N = 204)		
Surrogacy is a good way to help infertile couples have a child with their own genetic characteristics	Mean (SD)	3.92 (1.11)	3.62 (1.32)	3.79 (1.22)	0.028
Surrogacy reduces psychological tension in infertile couples	Mean (SD)	3.68 (1.11)	3.71 (1.26)	3.69 (1.18)	0.410
Surrogacy improves the life satisfaction of infertile couples	Mean (SD)	3.79 (1.08)	3.73 (1.22)	3.76 (1.14)	0.876
Surrogacy can prevent divorce and strengthens family structure	Mean (SD)	3.29 (1.14)	3.38 (1.25)	3.33 (1.19)	0.365
If there is no other infertility treatment option, surrogacy could be the last choice	Mean (SD)	3.80 (1.08)	3.80 (1.39)	3.80 (1.23)	0.185
I prefer to be voluntarily childless rather than to accept surrogacy	Mean (SD)	3.59 (1.29)	3.66 (1.47)	3.62 (1.38)	0.260
Adoption is better than surrogacy	Mean (SD)	2.73 (1.26)	2.96 (1.40)	2.83 (1.32)	0.060
Surrogacy is against religion	Mean (SD)	3.07 (1.18)	2.95 (1.43)	3.01 (1.30)	0.391

*N* frequency; % percentage

p < 0.05 is considered statistically significant

### Multivariate analysis

A multivariate analysis was conducted for each population alone and then for all participants in this study in order to find predictors of a higher acceptance of surrogacy. For this purpose, surrogacy scale total scores were categorized into 2 groups based on the mean score, below and above mean. The following variables were included in the multivariate analysis: age, sex, number of children, socioeconomic status, marital status (single, married, divorced), reported attitude toward surrogacy (supportive, neutral, against), awareness of any Lebanese couples who went for surrogacy as a solution for infertility, belief that surrogacy is worth discussing in Lebanon, knowledge about the religion’s position regarding surrogacy, and willingness to follow the religion’s position, and responses to all 3 clinical scenarios.

For medical students, the best predictors for increased acceptance of surrogacy were a supportive reported attitude toward surrogacy and the belief that surrogacy is a solution worth discussing in Lebanon. Statistically significant variables in the multivariate analysis for medical students are reported in Table 10.

**Table 10:** Multivariate analysis for medical students

	P value	OR	95% CI.for OR	
			Lower	Upper
What is your attitude toward surrogate pregnancy?	0.000	0.282	0.164	0.486
Do you believe surrogacy is a solution worth discussing in Lebanon as a potential treatment for infertility?	0.015	3.814	1.298	11.208

*OR* Odds Ratio; *CI* confidence interval

p < 0.05 is considered significant

For lawyers, the best predictors for increased acceptance of surrogacy were a younger age, not being single, a supportive reported attitude toward surrogacy, the belief that surrogacy is a solution worth discussing in Lebanon and not knowing the official position of their religion toward surrogate pregnancy. Statistically significant variables in the multivariate analysis for lawyers are reported in Table 11.

**Table 11:** Multivariate analysis for Lawyers

	P value	OR	95% CI.for OR	
			Lower	Upper
Age	0.041	0.234	0.058	0.940
Single	0.011	0.321	0.133	0.773
What is your attitude toward surrogate pregnancy?	0.000	0.068	0.034	0.137
Do you know the official position of your religion toward surrogate pregnancy?	0.030	0.380	0.158	0.913

*OR* odds ratio; *CI* confidence interval

p < 0.05 is considered significant

For both populations together, the best predictors for increased acceptance of surrogacy were not being single, a supportive reported attitude toward surrogacy and the belief that surrogacy is a solution worth discussing in Lebanon. Statistically significant variables in the multivariate analysis for both populations are reported in Table 12.

**Table 12:** Multivariate analysis for Both Populations

	P value	OR	95% C.I.for OR	
			Lower	Upper
Single	0.004	0.415	0.228	0.753
What is your attitude toward surrogate pregnancy?	0.000	5.464	3.65	8.13
Do you believe surrogacy is a solution worth discussing in Lebanon as a potential treatment for infertility?	0.002	4.186	1.709	10.256

*OR* odds ratio; *CI* confidence interval

p < 0.05 is considered significant

## Discussion

Our study illustrated the supportive attitude of medical students and lawyers toward surrogacy in Lebanon. Most lawyers are aware of the official stance of their religion regarding the topic while most medical students are not aware their religion’s official stance. Despite this difference, both lawyers and medical students relate their attitude to their social values rather than religion. Furthermore, medical students who are supportive of surrogacy believe that surrogacy is a solution for infertility in Lebanon. In addition to accepting surrogacy as a solution for infertility, supportive lawyers are young and not single.

Results from the current report contradict a recent study by Mustafa et al. [10] assessing the attitude of Jordanian medical and paramedical students toward surrogate pregnancy. In their report, 86.9% of participants were against surrogate pregnancy, 7.6% neutral, and 5.5% supportive compared to 10.1% against, 35.1% neutral, and 54.8% supportive in our population of medical students in our cohort. This can be explained by the discrepancy in the basis of decision making about the topic and the levels of religiosity in the two cohorts. While the majority (76.2%) of the Lebanese cohort based their decision on social values, 69.5% of the Jordanian cohort reported religion as the basis behind their attitude toward surrogacy. On the other hand, only 33.1% of Lebanese medical students know the official religious stance about the topic compared to 80.5% of medical students in the Jordanian study. Religiosity as a strong determinant of attitudes regarding surrogacy in Arabic speaking countries [18], and the different religious compositions in the two countries could further explain the more acceptive attitude toward surrogacy in Lebanon.

Accepting surrogacy as a viable option for people with infertility problems is a predictor of positive attitudes toward surrogacy in our cohort. Nearly all medical students and more than half of lawyers in our cohort are not married and are young adults with a median age of 22.5 and 36.6 respectively. This could translate into an increased acceptance of surrogacy by this population over time as some will unfortunately encounter infertility problems. For lawyers specifically, being not single was also associated with positive attitudes to surrogacy. This could also translate to an increased rate of acceptance to surrogacy in the following years as more people form this cohort will be married.

Some limitations of this study would include recall bias as this is a questionnaire-based study. Also, due to the barriers imposed by the COVID-19 pandemic, the sample was not designed to statistically represent the population of all lawyers in Lebanon and make rigid extrapolations, but to give sufficient insight, on the acceptance and attitude of some present and future decision makers regarding this highly debated topic worldwide. Also, the present study does not assess the attitudes about the implementation of altruistic surrogacy which can affect the response of the participants in the study. Finally, this study only included medical students and lawyers, and future studies will be needed to assess the perspective of attending physicians, statespersons, religious authorities, the general population, and most importantly, the perspective of infertile couples regarding this topic in Lebanon. To our knowledge this is the first study in Lebanon that assesses acceptance and attitude of medical students and lawyers regarding surrogate pregnancy while using a valid and reliable scale to assess acceptance of surrogacy in our populations.

## Conclusion

In conclusion, this study shows that only a minority of medical students and lawyers in Lebanon oppose the legislation of surrogate pregnancy. More studies are needed to explore the perspective of the general population especially that of people who benefited from surrogate pregnancy. Perspective of other stakeholders regarding this practice will also be of value to make better evidence-based decisions on a legislative level in Lebanon.

## Data Availability

The data supporting this study’s findings are available upon request from Dr. Rashad Nawfal.
